# Recent Advances in Protein–Protein Interactions

**DOI:** 10.3390/ijms24021282

**Published:** 2023-01-09

**Authors:** Igor A. Sedov, Yuriy F. Zuev

**Affiliations:** 1 Chemical Institute, Kazan Federal University, Kazan 420008, Russia; 2 FRC Kazan Scientific Center of RAS, Kazan Institute of Biochemistry and Biophysics, Kazan 420111, Russia

Protein-protein interactions (PPIs) lead to formation of complexes and aggregates between a pair or multiple protein molecules. They play a pivotal role in many processes in living organisms such as antibody–antigen binding, cell signaling, gene expression and control, viral self-assembly, and others. On the microscopic level, the PPIs are governed by multiple contributions from individual molecular fragments, which can be regarded as a combination of electrostatic, dispersion, hydrogen-bonding, hydrophobic, π-stacking interactions. PPIs are the objects of interest for several multidisciplinary fields of studies: proteomics, biophysics, biochemistry, bioinformatics, and pharmacology, which use their arsenal of tools to explore the links between structure, interactions, and functions of proteins and to design novel pharmaceuticals based on this knowledge.

This special issue contains 8 original and 4 review articles. The papers deal with different proteins from different organisms and use various experimental techniques as well as computational methods representing the diversity of research activities within the field.

Shoshani et al. [1] consider the challenging task to identify the binding partner of β_2_ subunit of human Na^+^, K^+^-ATPase mediating the adhesion of astrocytes to neurons. Surprisingly, their results allow to suggest that this protein is a homophilic cell-adhesion molecule when expressed on the plasma membrane of non-astrocytic cells such as fibroblasts or epithelial cells. This result raises a question about the existence of the self-avoiding mechanism of β_2_–β_2_ binding in astrocytes in a similar way to neurons avoiding the formation of synapses with themselves.

The paper of Eronina et al. [2] is dedicated to PPIs of a small human heat shock protein αB-crystallin and glycogen phosphorylase b. Chaperone activity of αB-crystallin leads to inhibition of the aggregation of glycogen phosphorylase b. The PPI changes the tertiary and quaternary structure of both the target protein and the chaperone. Authors show that the anti-aggregation activity increases in the presence of betaine (*N*,*N*,*N*-trimethylglycine) and decreases in the presence of arginine. Both ligands affect the stability of proteins and PPI strength.

In the work of Gorshkov et al. [3], diverse Svx-like proteins acting as virulence factors of phytopathogenic bacteria of the Pectobacterium genus are considered from the phylogenetic and functional points of view. The atomic model of Svx protein of *Pectobacterium atrosepticum* is considered, and its possible interactions with α-glycosylated proteins are discussed.

The contribution from Ilinskaya et al. [4] describes the differences between homologous RNases from various *Bacillus* species in their interactions with the inhibitor protein barstar and catalytic activity towards different RNA substrates. Even minor changes in the structure of considered proteins can cause significant effects on their stability and functional activity.

Anisenko et al. [5] analyze interactions of HIV-1 integrase and the cellular Ku70 protein, which is necessary for HIV replication. Using peptide fishing assay and site-directed mutagenesis, they identify the key residues of Ku70 for integrase binding. Then, the authors perform virtual screening of a library of small organic molecules in order to detect the ligands shielding these residues, which may disrupt the interactions between proteins and become prospective hits for the further development of novel anti-HIV drugs. Experimental testing of the top scoring compounds is also performed.

Mukhametzyanov et al. [6] study refolding of hen egg-white lysozyme in glycerol using a recently emerged method of fast scanning calorimetry. This method allows to work at millisecond timescales and provides a unique view of the non-equilibrium protein states.

In addition to the studies of particular proteins, two contributions deal with the analysis of the human protein–protein interactome. Cho et al. [7] report a comparative analysis of various network-based disease–gene association prediction methods. Some of these methods are based on graph-theoretic algorithms, others use machine learning techniques, and a few so-called integrative methods rely on both graph theory and machine learning. The authors evaluate the accuracy of disease–gene association prediction and determine that integrative approaches outperform other methods in the presence of known disease-associated genes. The advantages of using a heterogeneous network combining the human PPI network, disease network, and disease–gene associations as input are highlighted. Shepelyansky et al. [8] use the Google matrix algorithms for prediction of protein–protein interactions linked to myocardial fibrosis. They discover several potential fibrosis-associated proteins and identify the most important interactions between 54 proteins related to fibrotic cascade.

The review papers are also dedicated to the diverse experimental methods and proteins.

The contribution of Magnez et al. [9] describes microscale thermophoresis (MT) as a technique to study protein–protein as well as protein–ligand interactions. MT allows to quantify binding interactions by determining the binding constant values, offers the possibility of fast screening of large libraries, and may become the technology of choice in the drug discovery programs. Case studies of different PPIs using MT in order to detect therapeutically pertinent proteins in oncology, viral diseases and immuno-inflammatory pathologies are discussed.

Muronetz et al. [10] review various aspects of the influence of chaperones on amyloidogenic proteins. They consider both natural and artificial polymer-based chaperones. Blocking chaperones by misfolded proteins can cause increased formation of amyloid aggregates and pathological changes in the vital activity of cells, which occurs during the development of neurodegenerative diseases. Artificial chaperones might be used as anti-aggregation and anti-amyloid treatment compounds.

The paper of Kuznetsov et al. [11] summarizes the information on the effects of single nucleotide polymorphisms (SNPs) on DNA polymerase β structure and functions. Some of these SNPs can have dramatic consequences affecting the native fold of polymerase, its contacts with DNA, the catalytic activity, and interactions with other proteins.

Zuev et al. [12] consider the novel approach to extract the values of virial coefficients and the effective potential of pairwise protein–protein interactions from the experimental concentration dependencies of translational diffusion coefficients of rigid globular and partly disordered spheroidal proteins.
